# A Simple Index of Lake Ecosystem Health Based on Species-Area Models of Macrobenthos

**DOI:** 10.3390/ijerph19159678

**Published:** 2022-08-05

**Authors:** Junyan Wu, Yajing He, Yongjing Zhao, Kai Chen, Yongde Cui, Hongzhu Wang

**Affiliations:** 1 State Key Laboratory of Freshwater Ecology and Biotechnology, Institute of Hydrobiology, Chinese Academy of Sciences, Wuhan 430072, China; 2 University of Chinese Academy of Sciences, Beijing 100049, China; 3 State Key Laboratory of Marine Resource Utilization in South China Sea, Hainan University, Haikou 570228, China

**Keywords:** ecosystem health assessment, biological index, quantile regression model, shallow lakes, Yangtze River basin

## Abstract

An effective biological index should meet two criteria: (1) the selected parameters have clear relationships with ecosystem health and can be measured simply by standard methods and (2) reference conditions can be defined objectively and simply. Species richness is a widely used estimate of ecosystem condition, although it is increased by nutrient enrichment, a common disturbance. Based on macrobenthos data from 91 shallow Yangtze lakes disconnected from the mainstem, we constructed an observed species (*S_O_*)-area (*A*) model to predict expected species richness (*S_E_*), and then developed an observed to expected index (O/E-*_SA_*) by calculating the *S_O_*/*S_E_* ratio. We then compared O/E-*_SA_* with three other commonly used indices regarding their ability to discriminate cultivated and urban lakes: (1) River Invertebrate Prediction and Classification System (RIVPACS; O/E-*_RF_*), (2) Benthic Index of Biotic Integrity (B-IBI), and (3) Average Score Per Taxon (ASPT). O/E-*_SA_* showed significant positive linear relationships with O/E-*_RF_*, B-IBI and ASPT. Quantile regressions showed that O/E-*_SA_* and O/E-*_RF_* had hump-shape relationships with most eutrophication metrics, whereas B-IBI and ASPT had no obvious relationships. Only O/E-*_SA_*, O/E_50_ and B-IBI significantly discriminated cultivated from urban lakes. O/E-*_SA_* had comparable or higher performance with O/E-*_RF_*, B-IBI and ASPT, but was much simpler. Therefore, O/E-*_SA_* is a simple and reliable index for lake ecosystem health bioassessment. Finally, a framework was proposed for integrated biological assessment of Yangtze-disconnected lakes.

## 1. Introduction

Thousands of floodplain lakes (with a total area of 15,770 km^2^) are distributed along the mid-lower Yangtze River, playing very important roles in maintaining floodplain ecosystem functions. However, these lakes are seriously threatened by multiple stressors, including habitat loss, alterations of hydrological connectivity and flow regimes, water pollution (especially nutrient enrichment), and overexploitation of biological resources [1]. At present, official water body assessments of these lakes are mainly based on physico-chemical parameters as outlined in Environmental Quality Standards for Surface Water (GB 3838-2002) [2], which are incapable of reflecting ecosystem health comprehensively [3]. Therefore, there is an urgent need to construct biological indices reflecting the comprehensive impacts of stressors on these lakes. However, establishment and application of biological indices for freshwaters have so far mainly focused on streams and rivers [4,5,6], but few on lakes [7,8].

An effective biological index should meet two important criteria. First, the selected parameters should have clear relationships with ecosystem health, and the capacity to be measured simply using standard methods. Measurements of species richness and abundance of important taxa can meet this criterion [9,10,11], but widely used diversity indices (e.g., Shannon–Weiner diversity index) cannot. Such diversity indices have been formulated in various ways, but have a number of deficiencies. (1) They convey no information other than aspects of community composition and structure. (2) Different indices may indicate different trends in biodiversity or even reverse results. (3) Each diversity index attempts to synthesize information on species number and relative importance in some way, but the importance cannot be simply calculated as ratios of density, biomass, or production. (4) Different calculations can show quite different values. (5) Diversity indices often cannot reliably indicate the effects of environmental stressors [12,13,14,15]. Second, reference conditions should be able to be defined objectively and simply. The multi-metric method uses minimally disturbed sites, or least disturbed sites as references [16,17], but this lacks an objective criterion and is not suitable for unique natural and artificial ecosystems. The River Invertebrate Prediction and Classification System (RIVPACS) method determines reference conditions through a modelling approach [18,19], but the complicated modelling process generally requires a large set of reference biological samples from high quality, minimally disturbed sites, which makes it difficult to define reference conditions in many cases [20]. As such, these challenges have limited the widespread application of RIVPACS [21]. Hence, it is necessary to develop simple models to define reference conditions. Species-area models are simple and widely applicable [7,16,22,23] and can be used to determine expected values of species richness as reference condition. Thus, species–area models can be used to constructed bioassessment index.

Therefore, it is urgent to construct a simple and reliable biological index to assess the health condition of Yangtze floodplain lakes and provide a scientific basis for lake ecosystem restoration and protection. To establish a simple and reliable bioassessment system for Yangtze floodplain lakes disconnected from the mainstem, we used macrobenthos data in a three-step process. First, we defined reference conditions through use of quantile regression species–area models and constructed an observed to expected index based on a macrobenthos species–area model (O/E-*_SA_*). Then, we analyzed the effectiveness of O/E-*_SA_* by comparing its performance with common indices, including Benthic Index of Biotic Integrity (B-IBI), Average Score Per Taxon (ASPT), O/E indices based on Random Forest models (O/E-*_RF_*) and then assessed the relationships with eutrophication indicators. In the final step, we explain how to construct an integrated biological assessment of Yangtze-disconnected lakes.

## 2. Materials and Methods

### 2.1. Study Area and Data Sources

The study lakes cover a total area of more than 5400 km^2^, are located in the mid-lower Yangtze River Basin (28°30′ N~31°40′ N, 112°33′ E~121°00′ E), and have a warm and humid subtropical monsoon climate (Figure S1). The biological data in this study were collected by our research group during field surveys over the past 20 years (1998~2019). We compiled 116 lake-years data from 91 Yangtze mainstem-disconnected shallow lakes (1207 samples in total, Table S1). Sampling stations at each lake were systematically set in the offshore zones according to lake area, with the number of stations per lake ranging from 1 to 58. We analyzed the effects of sampling effort in Figure S2 to clarify that the sampling stations in each lake are appropriate and sufficient. All biological data selected for this study were quantitative measurements from spring and autumn to ensure comparability, except for a few lakes with only one season of the field survey.

All macrobenthos samples (1207 samples mentioned above) were sampled via one grab at each sampling station with a modified Peterson grab (1/16 m^2^), washed gently through a 425 μm sieve, and preserved in 10% formalin. After samples were rinsed with water in the laboratory, all individuals were sorted, counted, and identified to the lowest practical taxonomic level. Aquatic oligochaetes were identified to genus, polychaetes and leeches to family or genus, molluscs and arthropods to genus, and the remaining taxa to family [24,25]. Submersed macrophytes (B_Mac_) were sampled just above the sediment by scythes (1/5 m^2^) 2–4 replicates at each sampling point [26].

Physico-chemical parameters including water temperature, pH, conductivity, mean water depth (Z_M_), and Secchi depth (Z_SD_) were measured in situ during each visit. A water sample (1 L) was collected from each site during each visit and brought back to the laboratory to measure the concentrations of total nitrogen (TN), total phosphorus (TP), and chlorophyll *a* in phytoplankton (Chl *a*) [27]. We used the spring and autumn environmental parameters data to match with the biological data we used.

Landscape variables (climate, land cover) were extracted for each lake set to the World Geodetic System (WGS)-1984 Coordinate System and a grid resolution at 30 arc-second (ca. 1 km). We obtained mean air temperature and mean precipitation data (http://www.worldclim.org/, accessed on 30 December 2020) for each lake and land-cover information (http://www.globallandcover.com/, accessed on 30 December 2020) for a 500 m buffer along the lake shoreline, and classified the studied lakes as either urban or cultivated based on whether cultivated land or artificial surfaces dominated. We defined urban lakes when the proportion of artificial surfaces was >50% of the shoreline buffer, and we defined lakes as cultivated when the proportion of cultivated land was >50% of the shoreline buffer. Urban and cultivated lakes were used to test the discrimination power of indices. Landscape data were extracted using the zonal tool in ArcGIS 10.6.

### 2.2. Index Development

Four indices were developed: O/E-*_SA_* index, O/E-*_RF_* indices, B-IBI and ASPT (full name in Table S3). The O/E-*_SA_* index calculated expected species richness by species (*S_O_*)-area (*A*) modeling based on lake area and the observed species richness of each lake. O/E-*_RF_* indices were RIVPACS (River Invertebrate Prediction and Classification System) indices with species richness calculated by the sum of probabilities of capture (Pc) of taxa predicted by RF modeling. The constructed processes were as follows.

(1) O/E-*_SA_* index

A species (*S_O_*)-area (*A*) model was developed based on lake area to predict the expected species richness (*S_E_*) and calculated an observed to expected index (O/E-*_SA_*) by calculating the *S_O_*/*S_E_* ratio. We used percentage error (PE) to compare predictive power of three different linear regression forms, including a linear model (*S*/*A*), semi-log model (*S*/log_10_*A*), and power model (log_10_*S*/log_10_*A*). The formula was: PE = ∑|*P*/*O* − 1| × 100/*n*, where *P* is the expected value and *O* is the observed value [28]. Quantile regression models were developed to confirm the optimal and simpler model used to predict the expected species richness (*S_E_*). The optimal conditional quantiles were confirmed by model fitting (pseudo *R*^2^) and evaluation of two parameters (i.e., slope and intercept distribution). The biological condition of each lake was evaluated by calculating the ratio of the observed value (S*_O_*) to the expected value (S*_E_*).

(2) O/E-*_RF_* indices

O/E-*_RF_* indices were developed following established procedures [19,29,30]. First, we identified 21 reference lakes according to the status of lakes, available physico-chemical data, and professional judgment. These reference lakes (marked in Table S1) met the following conditions: (1) the lakeside zone was basically maintained in a natural state and the partial littoral area had submerged macrophytes, with an average biomass greater than 200 g/m^2^; (2) there was no or very little diffuse-source pollution around the lake; (3) there was no or little fishery disturbance (annual yield of fishery less than 15 t) than in other lakes. The pollution status was estimated qualitatively, and fishery disturbance was measured by the annual fishery yield, which ranged from 0 to 540 t. We then clustered reference lakes by applying the β-flexible clustering technique (β = −0.5) to pairwise Sørensen dissimilarities based on the presence and absence of macroinvertebrate taxa across the reference lakes. We then developed a RF model to predict cluster membership from natural environmental predictors (Table S4) and used the probabilities of cluster membership predicted by the RF model to weight taxon occurrence frequencies within reference site clusters to predict taxon-specific probabilities of capture (Pc). We calculated O/E based on taxa with Pc ≥ 0.5 (hereafter O/E_50_) and ≥0 (hereafter O/E_0_). In addition, we developed null O/E models with the Pc of individual taxa set to be equal across all sites. We used the randomForest package to develop Random Forest (RF) models with 1500 trees for each model [31].

(3) Other indices

We also calculated ASPT and B-IBI scores. The ASPT is based on the tolerance values of individual families to organic pollution [32]. The ASPT represents the average tolerance of organisms at the family level and can be determined by dividing the Biological Monitoring Party (BMWP) index score by the number of families present. The BMWP system also considers the tolerance of macroinvertebrates to organic pollution. Families are assigned a score between 1 and 10 according to their tolerances, then the BMWP score is the sum of the values for all families present in the sample [33]. The final ASPT score ranges between 1 and 6, the lower value represents higher tolerance of organic pollution (e.g., Oligochaeta have the highest tolerance and score as 1).

For B-IBI development [34], we started with 42 candidate metrics included in five metric categories: taxonomic richness, taxonomic composition, tolerance, functional feeding group, and habitat quality. Metrics were selected following range, discrimination power, and redundancy tests. First, metrics with a median of 0 for reference lakes were eliminated through range test. Second, the discrimination power of each metric was defined as the degree of inter-quartile overlap in the box plots of both reference and test sites for each metric. Third, metric redundancy was calculated using the Spearman correlation between all candidate metric. Metrics with high correlation (|r| > 0.7) and with *p* < 0.05 were removed. Finally, we selected the following four metrics to calculate the final score: total number of taxa, Biotic Index (BI), % Gastropoda individuals, and %collector-gatherer individuals. We used general taxa pollution tolerance values to calculate the Biotic Index (BI). We calculated the scores of metrics that decreased in response to stressors by the fraction of the 95th percentile value. We scored metrics that increased in response to stressors by the radio of the difference between the maximum value and the metric value and the difference between the maximum value and the 5th percentile value. The final B-IBI score were calculated by summing the scores of the four metrics.

Index performance of all indices was compared in terms of precision, bias, responsiveness, and sensitivity [35]. To facilitate performance comparisons among all indices, we calculated standardized O/E-*_SA_*, ASPT, and B-IBI scores by dividing raw scores by the mean of reference site scores so that reference site scores were centered on one. We used linear regression and Spearman rank correlation analysis to develop the relationship between each pair of all biological indices. We then compared the effectiveness of these indices by analyzing the relationship between macrobenthos indices and eutrophication metrics through quantile regression analysis. The discrimination power of urban and cultivated lakes by these indices was completed using a Wilcoxon test [36].

## 3. Results

### 3.1. Taxonomic Composition

Over the past 20 y, we collected a total of 188 macrobenthos taxa belonging to three phyla, 7sevenclasses, 48 families and 151 genera across all study lakes. The total number of taxa within each lake ranged from 2 to 42, with an average of 13 taxa per lake (Table S2; Figure S1). The most commonly collected taxa were *Limnodrilus* sp., *Branchiura sowerbyi*, *Bellamya* sp., *Chironomus* sp. and *Tanypus* sp., with taxa occurrence frequencies of 0.30, 0.41, 0.24, and 0.40, respectively.

### 3.2. O/E-_SA_ Modeling

**Figure 1:**
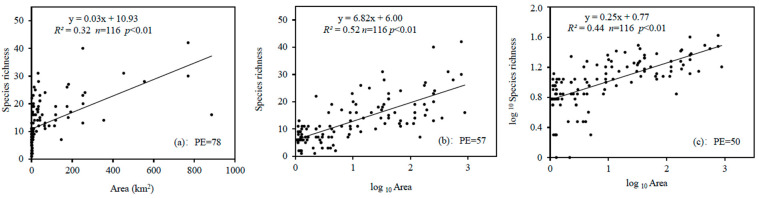
Species–area models for macrobenthos in shallow lakes of the mid-lower Yangtze River Basin. (**a**) linear regression (*S*/*A*), (**b**) semi-log model (*S*/log_10_*A*), and (**c**) power model (log_10_*S*/log_10_*A*).

In sampling effort analysis, the sampling point in lakes was adequate for local richness (*p* < 0.01) (Figure S2). In comparison with the linear regression (*S*/*A*, *R*^2^ = 0.32, PE = 78) (Figure 1a) and power models (log_10_*S*/log_10_*A*, *R*^2^ = 0.44, PE = 50) (Figure 1c), the semi-log model (*S*/log_10_*A*, *R*^2^ = 0.52, PE = 57) (Figure 1b) had the highest *R^2^* and lower PE than the linear regression. Therefore, the semi-log model was selected as the optimal model to estimate expected species richness.

**Figure 2:**
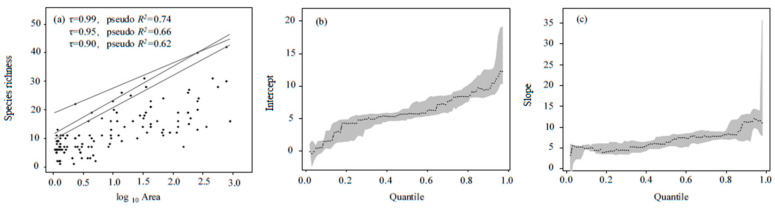
Quantile regression based on species–area relationships of macrobenthos (**a**) and its estimated intercept (**b**) and slope (**c**) (“τ” means the quantile of quantile regression, the gray areas represent 95% confidence intervals).

Based on the optimal model, we constructed quantile regression models (Figure 2). The fitting function at τ = 0.95 had the best performance, with a narrower 95% confidence interval for the intercept (9.6~17.6) and slope (9.8~14.2), and a higher pseudo *R*^2^ (0.66). Thus, we used this function to estimate the reference value. The formula was as follows:

*S_E_* = 11.9 log_10_*A* + 11.5

where *S_E_* is the expected value of species richness and *A* is lake area.

Then, the O/E-*_SA_* index was constructed with the following formula:

O/E-*_SA_* = *S_O_/S_E_*

where *S_O_* is the observed value of species richness. If the ratio was >1, O/E-*_SA_* = 1.

### 3.3. O/E-_RF_ Modeling

The performance of the modeled O/E_50_ was the best among these four indices (Table 1). We clustered 21 reference lakes based on the similarity of presence/absence macrobenthos data into four groups with each cluster group size ranging from four to eight (Table S1). The out-of-bag estimation-based accuracy of RF models for reference lakes was 86%. The results showed that the precision of O/E_50_ was much higher than that of O/E_0_. The accuracy of modeled indices with natural variation adjustment was higher than that of null indices with no natural variation adjustment. The modeled O/E_50_ had the best accuracy with bias of 0% variation among reference site values associated with natural variables after modeling. However, the other three models all had residual natural variation that could not be explained. All O/E-*_RF_* indices had high sensitivity, and there was little difference in their responsiveness.

### 3.4. Comparison of INDICES

#### 3.4.1. Relationships between O/E-*_SA_* and Other Indices

O/E-*_SA_* performed about the same as O/E_50_ in precision and sensitivity, but its bias was higher and its responsiveness was lower than that of O/E_50_ (Table 1). The O/E-*_SA_* also had better performance than B-IBI and ASPT in precision, bias, and sensitivity; however, both B-IBI and ASPT were more responsive than O/E-*_SA_*.

**Figure 3:**
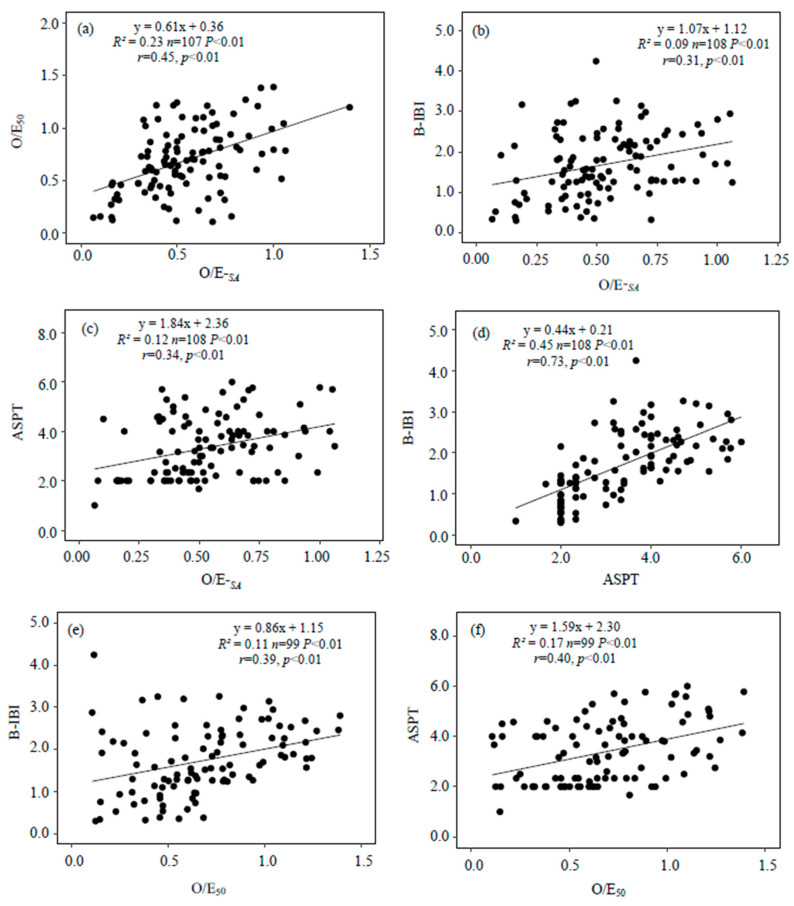
Relationships of O/E-*_SA_* with other indices ((**a**): O/E_50_, (**b**): B-IBI, (**c**): ASPT), B-IBI with ASPT (**d**), O/E_50_ with B-IBI (**e**) and ASPT (**f**) (“*r*” means coefficient of Spearman rank correlation).

Linear regression and Spearman rank correlation analysis indicated that O/E-*_SA_* had significant, but weak, positive correlations with O/E_50_, B-IBI, and ASPT (*r* = 0.45, 0.31, 0.34, *p* < 0.01) (Figure 3). The correlations of O/E_50_ with B-IBI and ASPT were also significantly and weakly positive (*r* = 0.39, 0.40, *p* < 0.01). However, the correlation of ASPT with B-IBI were moderately strong (*r* = 0.73).

#### 3.4.2. Relationships between Macrobenthos Indices and Eutrophication Metrics

**Figure 4:**
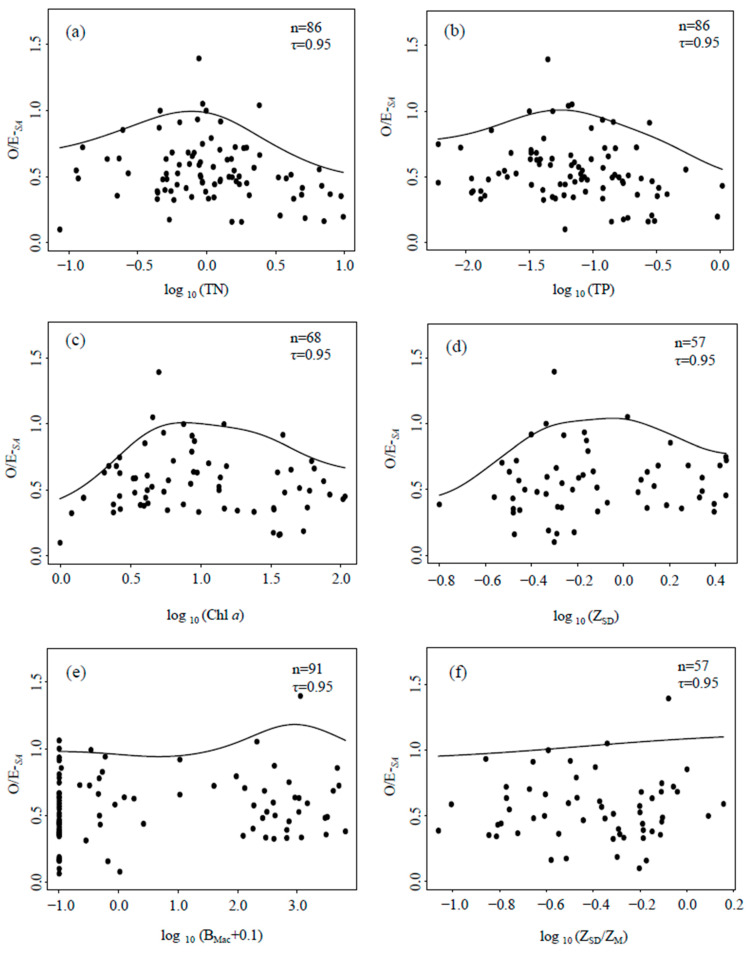
Quantile regressions of O/E-*_SA_* on total nitrogen (TN, mg/L) (**a**), total phosphorus (TP, mg/L) (**b**), phytoplankton chlorophyll *a* (Chl *a*, μg/L) (**c**), Secchi depth (Z_SD_, m) (**d**), submersed macrophytes biomass (B_Mac_, g/m^2^) (**e**), and the ratio of Secchi depth to water depth (Z_SD_/Z_M_, m) (**f**). (n differs because of environmental data deficient).

Quantile regressions (Figure 4) showed that O/E-*_SA_* had hump-shaped relationships with four eutrophication metrics (TN, TP, Chl *a* and Z_SD_), but had no obvious relationships with B_Mac_ or Z_SD_/Z_M_. O/E_50_ had similar relationships as those above (Figure S3), B-IBI scores declined with increased TP, but ASPT had no obvious relationships with eutrophication metrics (Figures S4 and S5).

#### 3.4.3. Comparison of Macrobenthos Index Scores in Cultivated and Urban Lakes

**Figure 5:**
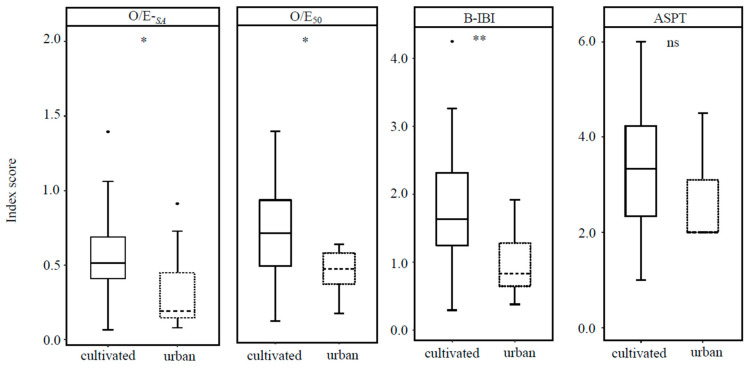
Box-plots of O/E-*_SA_*, O/E_50_, B-IBI, ASPT index scores in cultivated (solid line) and urban (dashed line) lakes. Boxes encompass the inter-quartiles, horizontal lines are medians, dots outside the box are outliers, and range bars show the maximum and minimum values excluding outliers. * = significance at *p* < 0.05 (Wilcoxon test) between index score in cultivated and urban lakes. ** = significance at *p* < 0.01. ns = no significant difference.

Figure 5 showed that O/E-*_SA_*, O/E_50_, and B-IBI (especially) significantly discriminated urban and cultivated dominant lakes, with higher index scores in the latter; however, ASPT scores did not do so.

## 4. Discussion

### 4.1. O/E-_SA_ Is a Simple and Reliable Index for Lake Biological Assessment

The rationale for applying O/E-*_SA_* is fourfold. (1) O/E-*_SA_* had a weakly significant positive correlation with the alternative indices (O/E_50_, B-IBI, and ASPT; Figure 3). (2) O/E-*_SA_* exhibited a unimodal hump-shaped relationship with eutrophication metrics (TN, TP, Chl *a*, and Z_SD_), which conforms to the common observation that species richness reaches a maximum at an intermediate level of nutrient enrichment [37]. However, O/E-*_SA_* had no obvious relationship with B_Mac_, which is likely related to the low collecting efficiency of Peterson grabs for epiphytic invertebrates. Previously, we found a positive correlation between the number of epiphytic gastropod taxa and the biomass of submerged macrophytes [26]. (3) O/E-*_SA_*, O/E-*_RF_* and B-IBI discriminated cultivated and urban lakes, but ASPT could not (Figure 5), and B-IBI scores declined with increased TP concentrations. Indices calculated by models (O/E-*_SA_*, O/E-*_RF_*) and based on multiple metrics (B-IBI) had better performance than ASPT, probably because ASPT is based on taxa tolerances to organic pollution versus nutrient enrichment. In addition, sensitive and tolerant taxa respond differently to disturbance [38], so in the future, we should construct separate species–area models for sensitive taxa and tolerant taxa to avoid equating increased species richness with greater ecosystem health. (4) O/E-*_SA_* had similar performance to O/E_50_, but was simpler to calculate.

Three standardized procedures should be developed when using O/E-*_SA_* for routine assessment. (1) We recommend using a grab sediment sampler (for example, Peterson grab, Van Veen grab) for sample collection. The grab sediment sampler used in this study is suitable for sediment collection in offshore zones. The collection of epiphytic benthos in littoral zones should be enhanced by sampling by hand and D-frame kick net [39,40]. (2) It is also important to define the sampling locations, number of sampling points, sampling frequency, and minimum number of specimens per lake to minimize effects of sampling effort on the number of taxa recorded [41,42]. (3) Specimens need to be identified to the lowest possible taxonomic level by identifiers with the same ability using standard keys [43]. It may be possible to simply identify specimens through DNA barcoding identification systems in the future [44,45], but we are not there yet. Following these standardized procedures, spatially extensive investigations should be carried out to rebuild the species–area model to set the expected value by lake type (cf. Figure 2).

### 4.2. Constructing an Integrated Biological Assessment Index for Lakes

**Figure 6:**
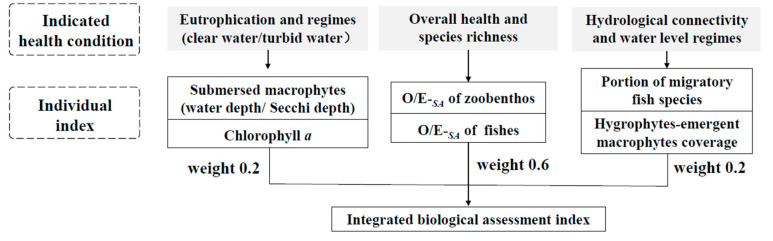
An integrated biological assessment index for Yangtze-disconnected floodplain lakes.

The main threats to Yangtze-disconnected floodplain lakes are eutrophication and hydrologic river–lake fragmentation [1]. Accordingly, we propose that an integrated ecological index should include three parts (Figure 6). (1) O/E-*_SA_* indices of biological assemblages (such as fishes and macrobenthos) to assess assemblage health conditions should have a weight of 0.3 each. For example, Whittier et al. (1997) and Whittier and Kincaid (1999) modeled fish species richness as a function of lake surface area and determined that non-native fish species reduced native fish species [46,47]. (2) The concentration of Chl *a* and the ratio of submersed macrophytes used to assess trophic state should have a weight of 0.2. The eutrophication process of a shallow lake can be largely described as a regime shift from a macrophyte-dominated clear-water state to an algae-dominated turbid-water state [48]. The water depth/Secchi depth correlates positively with submersed macrophyte biomass [26], so it can be used as a substitute when submersed macrophyte biomass data is deficient. (3) The portion of migratory species and hydrophyte-emergent macrophyte coverage to assess hydrologic conditions should have a weight of 0.2. The latter is closely related to water level fluctuations [23], and can be easily measured by remote sensing.

## 5. Conclusions

We constructed a simple and reliable index (O/E-*_SA_*) based on macrobenthos species richness to assess the biological condition of lake ecosystems, which was calculated from the ratio of the observed and expected values of species richness. The expected value of species richness was predicted through species (*S_O_*)-area (*A*) modeling. O/E-*_SA_* showed a significant positive linear relationship with commonly used indices and a hump-shaped relationship with eutrophication metrics. Therefore, O/E-*_SA_* is an effective index of lake biological health. It is worth noting that standardized procedures should be a concern in the application of this index. Furthermore, there still exist limitations, and an integrated index with multi-indicators may be better than an index based on single indicators. Thus, this modeled index approach should be applied to other biological assemblages and construct an integrated biological assessment index for Yangtze-disconnected lakes. 

## Figures and Tables

**Table 1:** Index performance based on precision, bias, sensitivity, and responsiveness.

	Reference Site			Test Site		
	Mean	Precision SD	Bias RF% Var	Mean	Sensitivity %NRC	Responsiveness *t*-Value
O/E_0_	0.99	0.33	9.52	0.54	1.00	6.96
O/E_0_-null	1.00	0.37	19.33	0.54	1.00	8.46
O/E_50_	1.02	0.18	0.00	0.61	0.99	8.29
O/E_50_-null	1.00	0.17	4.51	0.67	0.95	8.85
O/E-*_SA_*	1.00	0.20	0.42	0.74	0.99	3.94
B-IBI	1.00	0.26	3.53	0.64	0.94	5.23
ASPT	1.00	0.37	3.84	0.70	0.97	4.86

Precision (SD, standard deviation of reference values), bias (RF% Var, % of variation among reference site values associated with natural variables after modeling), sensitivity (%NRC, % of test considered as non-reference condition), and responsiveness (Student’s *t* value for the comparison between reference and index value of the most degraded sites).

## Data Availability

The data supporting the findings of this study are available within the article and the Supplementary Materials.

## Supplementary Materials

The following are available online at https://www.mdpi.com/article/10.3390/ijerph19159678/s1, Table S1: Basic information of the lakes. Table S2: Number of zoobenthos taxa in the five lake districts. Table S3: Abbreviation and full name of assessment indices. Table S4: Abbreviation and description of potential environmental predictors used in modeling. Figure S1. Spatial pattern of macrobenthos of shallow lakes along the mid-lower Yangtze River. Figure S2. Sampling point vs. residual local taxa richness for macrozoobenthos. Figure S3. Quantile regressions of O/E_50_ index on total nitrogen (TN, mg/L), total phosphorus (TP, mg/L), chlorophyll *a* of phytoplankton (Chl *a*, μg/L), Secchi depth (Z_SD_, m), annual submersed macrophytes biomass (B_Mac_, g/m^2^) and ratio of Secchi depth to water depth (Z_SD_/Z_M_, m) (sample sizes differ because of deficient sampling). Figure S4. Quantile regressions of B-IBI index on total nitrogen (TN, mg/L), total phosphorus (TP, mg/L), chlorophyll *a* of phytoplankton (Chl *a*, μg/L), Secchi depth (Z_SD_, m), annual submersed macrophytes biomass (B_Mac_, g/m^2^) and ratio of Secchi depth to water depth (Z_SD_/Z_M_, m) (sample sizes differ because of deficient sampling). Figure S5. Quantile regressions of ASPT index on total nitrogen (TN, mg/L), total phosphorus (TP, mg/L), chlorophyll *a* of phytoplankton (Chl *a*, μg/L), Secchi depth (Z_SD_, m), annual submersed macrophytes biomass (B_Mac_, g/m^2^) and ratio of Secchi depth to water depth (Z_SD_/Z_M_, m) (sample sizes differ because of deficient sampling).

## References

[1] Wang H.Z., Liu X.Q., Wang H.J., Chen Y., Chapman D.C., Jackson J.R., Chen D., Li Z., Killgore K.J., Phelps Q., Eggleton M.A. (2016). The Yangtze River Floodplain: Threats and Rehabilitation. Fishery Resources, Environment, and Conservation in the Mississippi and Yangtze.

[2] SEPA, AQSIQ (2002). Environmental Quality Standards for Surface Water: GB3838–2002.

[3] Hughes R.M., Noss R.F. (1992). Biological Diversity and Biological Integrity: Current Concerns for Lakes and Streams. Fisheries.

[4] Chen K. (2019). Incorporating functional traits to enhance multimetric index performance and assess land use gradients. Sci. Total Environ..

[5] Klemm D.J., Blocksom K.A., Fulk F.A., Herlihy A.T., Hughes R.M., Kaufmann P.R., Peck D.V., Stoddard J.L., Thoeny W.T., Griffith M.B. (2003). Development and evaluation of a Macroinvertebrate Biotic Integrity Index (MBII) for regionally assessing Mid-Atlantic Highlands Streams. Environ. Manag..

[6] Ruaro R., Gubiani É.A., Hughes R.M., Mormul R.P. (2020). Global trends and challenges in multimetric indices of biological condition. Ecol. Indic..

[7] Beck M.W., Hatch L.K. (2009). A review of research on the development of lake indices of biotic integrity. Environ. Rev..

[8] Kuehne L.M., Olden J.D., Strecker A.L., Lawler J.J., Theobald D.M. (2017). Past, present, and future of ecological integrity assessment for fresh waters. Front. Ecol. Environ..

[9] Albrecht J., Peters M.K., Becker J.N., Behler C., Classen A., Ensslin A., Ferger S.W., Gebert F., Gerschlauer F., Helbig-Bonitz M. (2021). Species richness is more important for ecosystem functioning than species turnover along an elevational gradient. Nat. Ecol. Evol..

[10] Gaston K. (2000). Global patterns in biodiversity. Nature.

[11] Gotelli N.J., Colwell R.K. (2001). Quantifying biodiversity: Procedures and pitfalls in the measurement and comparison of species richness. Ecol. Lett..

[12] Cairns J., McCormick P.V., Niederlehner B.R. (1993). A proposed framework for developing indicators of ecosystem health. Hydrobiologia.

[13] De Jonge M., Van de Vijver B., Blust R., Bervoets L. (2008). Responses of aquatic organisms to metal pollution in a lowland river in Flanders: A comparison of diatoms and macroinvertebrates. Sci. Total Environ..

[14] Hurlbert S.H. (1971). The Nonconcept of Species Diversity: A Critique and Alternative Parameters. Ecology.

[15] Koperski P., Meronka R. (2017). Environmental quality of a stream can be better predicted by phylogenetic than by taxonomic diversity. Knowl. Manag. Aquat. Ecosyst..

[16] Stoddard J.L., Larsen D.P., Hawkins C.P., Johnson R.K., Norris R.H. (2006). Setting expectations for the ecological condition of running waters the concept of reference condition. Ecol. Appl..

[17] Hering D., Feld C.K., Moog O., Ofenbock T. (2006). Cook book for the development of a Multimetric Index for biological condition of aquatic ecosystems: Experiences from the European AQEM and STAR projects and related initiatives. Hydrobiologia.

[18] Clarke R.T., Wright J.F., Furse M.T. (2003). RIVPACS models for predicting the expected macroinvertebrate fauna and assessing the ecological quality of rivers. Ecol. Model..

[19] Hawkins C.P., Norris R.H., Hogue J.N., Feminella J.W. (2000). Development and evaluation of predictive models for measuring the biological integrity of streams. Ecol. Appl..

[20] Clarke R.T., Murphy J.F. (2006). Effects of locally rare taxa on the precision and sensitivity of RIVPACS bioassessment of freshwaters. Freshwat. Biol..

[21] Chen K., Chen Q.W., YU H.Y., Wang B.X., Jin X.W., Wang Y.Y., Xu R.J., Cai K. (2018). Methods and prospects of index of biological integrity used for China river ecological health assessment. China Environ. Sci..

[22] Fausch K.D., Karr J.R., Yant P.R. (1984). Regional Application of an Index of Biotic Integrity Based on Stream Fish Communities. Trans. Am. Fish. Soc..

[23] Liu X., Yang Z., Yuan S., Wang H. (2017). A novel methodology for the assessment of water level requirements in shallow lakes. Ecol. Eng..

[24] Morse J.C., Yang L.F., Tian L.X. (1994). Aquatic Insects of China Useful for Monitoring Water Quality.

[25] Wang H.Z. (2002). Studies on Taxonomy, Distribution and Ecology of Microdrile Oligochaetes of China, with Description of Two New Species from the Vicinity of the Great Wall Station of China, Antarctica.

[26] Wang H.J., Pan B.Z., Liang X.M., Wang H.Z. (2006). Gastropods on Submersed Macrophytes in Yangtze Lakes: Community Characteristics and Empirical Modelling. Int. Rev. Hydrobiol..

[27] Huang X.F., Chen W.M., Cai Q.M. (1999). Survey, Observation and Analysis of Lake Ecology.

[28] Canfield D.E.J. (1983). Prediction of chlorophyll a concentrations in Florida lakes: The importance of phosphorus and nitrogen. Water Resour. Bull..

[29] Hawkins C.P., Carlisle D.M. (2001). Use of Predictive Models for Assessing the Biological Integrity of Wetlands and Other Aquatic Habitats. Bioassessment and Management of North American Freshwater Wetlands.

[30] Van Sickle J., Hawkins C.P., Larsen D.P., Herlihy A.T. (2005). A null model for the expected macroinvertebrate assemblage in streams. J. N. Am. Benthol. Soc..

[31] Liaw A., Wiener M. (2002). Classification and Regression by randomForest. R News.

[32] Armitage P.D., Moss D., Wright J.F., Furse M.T. (1983). The performance of a new biological water quality score system based on macroinvertebrates over a wide range of unpolluted running-water sites. Water Res..

[33] Zhang J.W., Cai K., Yu H.Y., Jiang Y.W., Li X.W., Zhou S.L., Xie Z.C., Wang Y.Y., Jin X.W., Wang B.X. (2018). Establishment of Chinese Macroinvertebrate Score Index and Water Quality Boundary. Environ. Monit. China.

[34] Barbour M.T., Gerritsen J., Griffith G.E., Frydenborg R., McCarron E., White J.S., Bastian M.L. (1996). A framework for biological criteria for Florida streams using benthic macroinvertebrates. J. N. Am. Benthol. Soc..

[35] Hawkins C.P., Cao Y., Roper B. (2010). Method of predicting reference condition biota affects the performance and interpretation of ecological indices. Freshw. Biol..

[36] Lemm J.U., Feld C.K., Birk S. (2019). Diagnosing the causes of river deterioration using stressor-specific metrics. Sci. Total Environ..

[37] Dodson S.I., Arnott S.E., Cottingham K.L. (2000). The relationship in lake communities between primary productivity and species richness. Ecology.

[38] Karr J.R. (1981). Assessment of Biotic Integrity Using Fish Communities. Fisheries.

[39] Heatherly T., Whiles M.R., Knuth D., Garvey J.E. (2005). Diversity and community structure of littoral zone macroinvertebrates in southern Illinois reclaimed surface mine lakes. Am. Midl. Nat..

[40] Porst G., Bader S., Munch E., Pusch M. (2012). Sampling approaches for the assessment of shoreline development based on littoral macroinvertebrates: The case of Lake Werbellin, Germany. Fundam. Appl. Limnol..

[41] Birk S., Bonne W., Borja A., Brucet S., Courrat A., Poikane S., Solimini A., van de Bund W., Zampoukas N., Hering D. (2012). Three hundred ways to assess Europe’s surface waters: An almost complete overview of biological methods to implement the Water Framework Directive. Ecol. Indic..

[42] Chen K., Hughes R.M., Wang B. (2015). Effects of fixed-count size on macroinvertebrate richness, site separation, and bioassessment of Chinese monsoonal streams. Ecol. Indic..

[43] Borja A., Tunberg B.G. (2011). Assessing benthic health in stressed subtropical estuaries, eastern Florida, USA using AMBI and M-AMBI. Ecol. Indic..

[44] Hering D., Borja A., Jones J.I., Pont D., Boets P., Bouchez A., Bruce K., Drakare S., Hanfling B., Kahlert M. (2018). Implementation options for DNA-based identification into ecological status assessment under the European Water Framework Directive. Water Res..

[45] Pawlowski J., Kelly-Quinn M., Altermatt F., Apotheloz-Perret-Gentil L., Beja P., Boggero A., Borja A., Bouchez A., Cordier T., Domaizon I. (2018). The future of biotic indices in the ecogenomic era: Integrating (e)DNA metabarcoding in biological assessment of aquatic ecosystems. Sci. Total Environ..

[46] Whittier T.R., Halliwell D.B., Paulsen S.G. (1997). Cyprinid distributions in Northeast USA lakes: Evidence of regional-scale minnow biodiversity losses. Can. J. Fish. Aquat. Sci..

[47] Whittier T.R., Kincaid T.M. (1999). Introduced Fish in Northeastern USA Lakes: Regional Extent, Dominance, and Effect on Native Species Richness. Trans. Am. Fish. Soc..

[48] Wang H.J., Wang H.Z., Liang X.M., Wu S.K. (2014). Total phosphorus thresholds for regime shifts are nearly equal in subtropical and temperate shallow lakes with moderate depths and areas. Freshw. Biol..

