# Supplementary material for: A Simple Index of Lake Ecosystem Health Based on Species-Area Models of Macrobenthos
Source: Int J Environ Res Public Health. 2022 Aug 5;19(15):9678. doi: 10.3390/ijerph19159678 (PMC9367816; doi:10.3390/ijerph19159678)
Supplement: Supplementary file 1 [file ijerph-19-09678-s001.zip › ijerph-1803582-supplementary.pdf]

Supplementary Material:

Table S1: Basic information of the lakes.

Table S2: Number of zoobenthos taxa in the five lake districts.

Table S3: Abbreviation and full name of assessment indices

Table S4: Abbreviation and description of potential environmental predictors used in modeling.

Figure S1. Spatial pattern of macrobenthos of shallow lakes along the mid-lower Yangtze River.

Figure S2. Sampling point vs. residual local taxa richness for macrozoobenthos.

Figure S3. Quantile regressions of O/E_{50} index on total nitrogen (TN, mg/L), total phosphorus (TP, mg/L), chlorophyll *a* of phytoplankton (Chl *a*, μg/L), Secchi depth (Z_{SD}, m), annual submersed macrophytes biomass (B_{Mac}, g/m^2) and ratio of Secchi depth to water depth (Z_{SD}/Z_M, m) (sample sizes differ because of deficient sampling).

Figure S4. Quantile regressions of B-IBI index on total nitrogen (TN, mg/L), total phosphorus (TP, mg/L), chlorophyll *a* of phytoplankton (Chl *a*, μg/L), Secchi depth (Z_{SD}, m), annual submersed macrophytes biomass (B_{Mac}, g/m^2) and ratio of Secchi depth to water depth (Z_{SD}/Z_M, m) (sample sizes differ because of deficient sampling).

Figure S5. Quantile regressions of ASPT index on total nitrogen (TN, mg/L), total phosphorus (TP, mg/L), chlorophyll *a* of phytoplankton (Chl *a*, μg/L), Secchi depth (Z_{SD}, m), annual submersed macrophytes biomass (B_{Mac}, g/m^2) and ratio of Secchi depth to water depth (Z_{SD}/Z_M, m) (sample sizes differ because of deficient sampling).

Supplementary Material

Table S1. Basic information of the lakes

ID	Lake names	Latitude	Longitude	Water area (km^2)	Shoreline _development	Z_{Mean}(m)	Z_M (m)	Z_{SD} (m)
1	Central Taihu Lake	120°04′-120°18′E	31°01′-31°19′N	885.00	3.2	2.2	2.30	0.35
2	Chaohu Lake1	117°16′-117°51′E	31°25′-31°43′N	769.55	1.92	2.6	1.56	0.40
3	Chaohu Lake2	117°16′-117°51′E	31°25′-31°43′N	769.55	1.92	2.6		
4	Southwest Taihu Lake	119°54′-120°14′E	30°56′-31°18′N	555.00	3.2	2.2	1.92	0.32
5	North Taihu Lake	119°58′-120°24′E	31°19′-31°32′N	453.00	3.2	2.2	2.03	0.34
6	East Area of Taihu Lake[*1]	120°18′-120°29′E	31°02′-31°18′N	263.00	3.2	2.2	1.69	0.46
7	Longganhu Lake1[*1]	115°59′-116°18′E	29°51′-30°05′N	252.00	4.88	5.5		
8	Longganhu Lake2	115°59′-116°18′E	29°51′-30°05′	252.00	4.88	5.5	1.99	1.19
9	Longganhu Lake3	115°59′-116°18′E	29°51′-30°05′N	253.00	4.88	5.5	1.65	0.37
10	East Taihu Lake[*1]	120°20′-120°38′E	30°56′-31°12′N	182.00	3.2	2.2	1.50	0.30
11	Pohu Lake	116°19′-116°33′E	30°04′-30°15′N	180.40	4.05	5.7		
12	Liangzihu Lake[*1]	114°25′-114°36′E	30°07′-30°17′N	174.52	8.8	7.4	2.23	1.42
13	Huanghu Lake1[*1]	116°23′-116°32′E	29°56′-30°05′N	118.60	4.58	6		
14	Huanghu Lake2	116°23′-116°32′E	29°56′-30°05′N	118.60	4.58	6	1.74	0.27
15	Huanghu Lake3	116°23′-116°32′E	29°56′-30°05′N	118.60	4.58	6	1.80	0.16
16	Futouhu Lake	114°09′-114°20′E	29°55′-30°07′N	52.60	4.87	5.2	0.50	0.60
17	Niushanhu Lake1[*1]	114°27′-114°38′E	30°16′-30°22′N	40.81	8.8	7.4	2.90	2.63
18	Niushanhu Lake2	114°27′-114°38′E	30°16′-30°22′N	40.81	8.8	7.4	2.46	1.91
19	Donghu Lake	114°21′-114°28′E	30°31′-30°36′N	33.70	3.405	4	2.92	0.74
20	Dongtangxunhu Lake1[*1]	114°19′-114°29′E	30°23′-30°29′N	31.75	4.92	4.1	2.28	1.04
21	Dongtangxunhu Lake2	114°19′-114°29′E	30°23′-30°29′N	31.75	4.92	4.1	1.60	0.67
22	Chenhu Lake	113°50′-113°52′E	30°17′-30°20′N	11.10	2.89	1		
23	Cihu Lake	115°01′-115°05′E	30°11′-30°13′N	10.50	2.47	8.5	2.32	0.51

24	Zhangjiadahu Lake	113°48′-113°50′E	30°17′-30°17′N	8.11	2.89	1	0.93	0.35
25	Honghu Lake[*2]	113°11′-113°28′E	29°38′-29°59′N	355.00	5.34	2.2		
26	Longganhu Lake[*2]	115°59′-116°18′E	29°51′-30°05′N	252.00	4.88	5.5	0.71	0.33
27	Junshanhu Lake[*2]	116°15′-116°28′E	28°24′-28°38′N	192.50	5.62	7.5	3.37	2.14
28	Dianshanhu Lake	120°53′-120°01′E	31°04′-31°12′N	63.70	1.86	1.8	1.93	0.54
29	Luhu Lake[*3]	114°08′-114°17′E	30°09′-30°20′N	29.80	4.04	3.1	2.61	1.16
30	Tian’e zhou Oxbow	112°33′-112°37′E	29°47′-29°51′N	20.00	2.92	2.6	7.94	0.64
31	Laojianghe Oxbow	112°59′-113°04′E	29°31′-29°35′N	18.40	2.95	1.8	5.30	0.68
32	Huamahu Lake[*4]	114°58′-115°04′E	30°15′-30°22′N	10.30	4	3.55	2.18	0.70
33	Nanhu Lake	114°12′-114°18′E	30°27′-30°31′N	7.67	3.29	4.3	2.36	0.33
34	Taojiadahu Lake[*4]	114°36′-114°38′E	30°37′-30°39′N	3.00	4.75	4.9	1.69	2.20
35	Sanliqihu Lake	114°56′-114°57′E	30°05′-30°06′N	2.70	2.07	2.3	1.95	0.51
36	Qinglinghu Lake	114°12′-114°16′E	30°22′-30°27′N	2.00	2.45	2.9	1.28	0.46
37	Moshuihu Lake	114°11′-114°15′E	30°31′-30°34′N	1.50	2.61	2.1	2.19	0.33
38	Qihu Lake[*4]	114°37′-114°40′E	30°36′-30°37′N	1.30	2.41	2.5		
39	Hongxinghu Lake	114°57′-114°57′E	30°05′-30°05′N	0.50	1.08	2.9	2.73	0.52
40	Main Area of Baoanhu Lake1	114°39′-114°49′E	30°12′-30°18′N	18.00	3.54	3.4		
41	Xiaosihai Lake1	114°40′-114°42′E	30°17′-30°18′N	7.00	3.54	3.4		
42	Shahu Lake	114°19′-114°21′E	30°33′-30°34′N	3.08	1.49	3.7	0.50	0.34
43	Yanxihu Lake[*1]	114°27′-114°30′E	30°32′-30°35′N	14.23	4.34	3.9	3.10	0.54
44	Yandonghu Lake[*4]	114°32′-114°35′E	30°31′-30°33′N	9.11	2.6	4.4	1.60	1.60
45	Shuiguohu Lake	114°21′-114°21′E	30°33′-30°33′N	0.12	4.55	5.8	2.50	0.55
46	Yangchunhu Lake	114°25′-114°25′E	30°37′-30°37′N	2.00	2.21	2.1	0.80	0.50
47	Beihu Lake1	114°30′-114°31′E	30°36′-30°37′N	3.00	1.37	1.6	2.00	0.61
48	Zhuzihu Lake	114°31′-114°32′E	30°34′-30°34′N	7.00	1.52	2.4		
49	Zhangduhu Lake	114°40′-114°48′E	30°37′-30°42′N	35.20	1.29	1.4	1.67	0.85
50	Gehu Lake	119°44′-119°53′E	31°29′-31°42′N	146.50	1.52	2.9	0.94	0.47
51	Yangchenghu Lake	120°39′-120°51′E	31°21′-31°30′N	113.00	3.8	1.7	1.45	0.77

52	Wuchanghu Lake[*2]	116°36′-116°53′E	30°14′-30°20′N	86.60	3.275	3.95		
53	Longyanghu Lake1	114°09′-114°12′E	30°32′-30°34′N	1.80	2.33	2.2		
54	Longyanghu Lake2	114°09′-114°12′E	30°32′-30°34′N	1.80	2.33	2.2		
55	Niushanhu Lake3	114°27′-114°38′E	30°16′-30°22′N	40.81	8.8	7.4	3.57	2.79
56	Niushanhu Lake4	114°27′-114°38′E	30°16′-30°22′N	40.81	8.8	7.4	3.57	2.79
57	West Liangzihu Lake1	114°25′-114°29′E	30°09′-30°14′N	66.70	8.8	7.4	3.79	2.48
58	West Liangzihu Lake2	114°25′-114°29′E	30°09′-30°14′N	66.70	8.8	7.4	3.79	2.48
59	Main Area of Baoanhu Lake2	114°39′-114°49′E	30°12′-30°18′N	23.90		3.4	2.08	1.79
60	Biandantang Lake1	114°43′-114°44′E	30°17′-30°18′N	3.30		3.4	2.17	1.36
61	Xiaosihai Lake2	114°40′-114°42′E	30°17′-30°18′N	1.30		3.4	1.80	1.27
62	Baoankou Lake1[*3]	114°43′-114°44′E	30°12′-30°13′N	2.80	3.54	3.4	2.18	1.54
63	Qiaodunhu Lake1	114°39′-114°41′E	30°14′-30°15′N	8.00	3.54	3.4		
64	Qiaodunhu Lake2	114°39′-114°41′E	30°14′-30°15′N	8.00		3.4	1.90	0.42
65	Baoankou Lake1	114°43′-114°44′E	30°12′-30°13′N	2.80		3.4	1.70	0.69
66	Biandantang Lake2	114°43′-114°44′E	30°17′-30°18′N	3.30	3.54	3.4	1.80	0.46
67	Xiaosihai Lake3[*3]	114°40′-114°42′E	30°17′-30°18′N	1.30		3.4	0.60	0.50
68	Niushanhu Lake5	114°27′-114°38′E	30°16′-30°22′N	40.81		3.4	2.80	2.20
69	West Liangzihu Lake3[*3]	114°25′-114°29′E	30°09′-30°14′N	66.70		3.4	3.20	2.80
70	Houguanhu Lake1	114°06′-114°07′E	30°29′-30°30′N	12.70	4.27	2		
71	Zhushanhu Lake	114°05′-114°08′E	30°26′-30°27′N	3.69	4.79	2.8		
72	Chuanjiangchi Lake	114°07′-114°08′E	30°25′-30°25′N	0.29	2.22	2.1		
73	Guanlianhu Lake1	114°01′-114°04′E	30°23′-30°24′N	5.39	3.13	2.8		
74	Zhongshanhu Lake	114°05′-114°05′E	30°24′-30°24′N	0.20	1.86	2.3		
75	Nantaizihu Lake	114°11′-114°12′E	30°29′-30°30′N	3.57	1.71	2.6		
76	Wanjiahu Lake	114°11′-114°12′E	30°28′-30°28′N	1.05	1.64	2.4		
77	Zhulinhu Lake	114°09′-114°10′E	30°26′-30°26′N	0.23	1.64	1.8		
78	Wulanghu Lake	114°05′-114°05′E	30°24′-30°24′N	0.31	1.99	2.1		
79	Longhu Lake	114°08′-114°08′E	30°22′-30°22′N	0.09	1.64	1.8		

80	Tanzihu Lake	114°00′-114°00′E	30°20′-30°20′N	0.17		4		
81	Wanhu Lake	114°01′-114°01′E	30°21′-30°21′N	0.24	1.12	1.4		
82	Shentanhu Lake	114°00′-114°00′E	30°20′-30°20′N	0.11		1.7		
83	Xiashanhu Lake	114°01′-114°01′E	30°21′-30°21′N	0.23		1.7		
84	Zhumuhu Lake	114°05′-114°05′E	30°22′-30°22′N	0.12	1.49	2.1		
85	Niuweihu Lake	114°05′114°05′E	30°24′-30°24′N	0.05		1.5		
86	Shangwuqiu Lake	114°05′-114°05′E	30°24′-30°24′N	0.12		1.4		
87	Zhuangyuanhu Lake	114°05′-114°05′E	30°24′-30°24′N	0.23	2.22	2.1		
88	Houguanhu Lake2	114°06′-114°07′E	30°29′-30°30′N	12.70	4.27	2	2.52	1.27
89	Sanjiaohu Lake	114°10′-114°11′E	30°31′-30°32′N	0.50	1.72	2.1	1.60	0.77
90	Liangzihu Lake2	114°25′-114°36′E	30°07′-30°17′N	174.52		3.4		
91	Houguanhu Lake3	114°00′-114°07′E	30°28′-30°33′N	34.00	4.32	2.2		
92	Wuhu Lake	114°28′-114°32′E	30°46′-30°50′N	30.60	2.81	2.5		
93	Wangjiashe Lake*4	113°52′-113°53′E	30°20′-30°21′N	8.75	1.34	0.5		
94	Guanlianhu Lake2	114°01′-114°04′E	30°23′-30°24′N	5.39	3.13	2.8		
95	Chaibohu Lake	114°34′-114°35′E	30°40′-30°42′N	3.30	1.9	2.8		
96	Beihu Lake2	114°30′-114°32′E	30°35′-30°37′N	1.94	1.37	1.6		
97	Longyanghu Lake	114°09′-114°12′E	30°32′-30°34′N	1.68	2.33	2.2		
98	Pingtanghu Lake	114°08′-114°08′E	30°18′-30°19′N	1.65	3.18	3.2		
99	Jinduihu Lake	113°58′-113°58′E	30°23′-30°24′N	0.68	1.66	2.5		
100	Qingtanhu Lake	114°32′-114°34′E	30°33′-30°34′N	0.60	1.91	3.6		
101	Longjiadahu Lake	113°53′-113°54′E	30°26′-30°27′N	0.57	2.09	2.1		
102	Wanglanghu Lake	114°08′-114°08′E	30°18′-30°18′N	0.43	1.44	1.5		
103	Chongrenhu Lake	113°56′-113°56′E	30°25′-30°26′N	0.38	1.59	2.4		
104	Biyanhu Lake	113°55′-113°55′E	30°26′30°26′N	0.31		1.5		
105	Xiaoguanlianhu Lake	114°03′-114°04′E	30°22′30°22′N	0.29	1.42	2.1		
106	Yangjianghu Lake	114°07′-114°08′E	30°19′-30°20′N	0.29		1		
107	Qianhu Lake	114°09′114°09′E	30°16′-30°16′N	0.19		1		
108	Daoshihu Lake	114°11′-114°11′E	30°23′-30°23′N	0.16	3.47	1.3		

109	Yanjiahu Lake	114°35′-114°35′E	30°38′-30°39′N	0.15	4.75	4.9
110	Xiaojinjishai Lake	113°55′-113°55′E	30°26′-30°26′N	0.13	2.49	1.8
111	Guojiahu Lake	114°11′-114°11′E	30°22′-30°22′N	0.12	1.3	2.6
112	Zhangjiadahu Lake2	113°48′-113°50′E	30°17′-30°17′N	8.11	2.89	1
113	Xiashehu Lake	114°09′-114°10′E	30°08′-30°08′N	1.17	2.05	2
114	Songjiaqi Lake	114°10′-114°10′E	30°10′-30°10′N	0.28	1.65	2
115	Lianhuahu Lake	114°16′-114°17′E	30°33′-30°33′N	0.28		1
116	Kuzhuhai Lake	114°12′-114°13′E	30°05′-30°06′N	7.25	1.62	1.5

*Reference site used in O/E-*RF*; number after *represents reference group used in O/E-*RF*; Z_{Mean}, mean water depth; the lakes are ordered by the data sources; the numbers immediately after the lake names mean different year visited.

Table S2. Number of zoobenthos taxa in the five lake districts (1998-2019)

	Taihu Plain	Suwan Plain	Ganwan Plain	Jianghan Plain	Dongtinghu Plain	Total	Percent (%)
**Annelida**							
**Oligochaeta**	12	9	6	15	23	25	13.3
Naididae	12	9	6	15	23	25	13.3
**Hirudinea**	1	0	2	7	0	8	4.3
Erpobdellidae	0	0	0	2	0	2	1.1
Glossiphoniidae	1	0	2	5	0	6	3.2
Hirudinidae	0	0	0	1	0	1	0.5
**Polychaeta**	5	3	2	0	0	6	3.2
Capitellidae	1	1	0	0	0	1	0.5
Nephtyidae	1	1	1	0	0	2	1.1
Nereididae	1	1	1	0	0	1	0.5
Sabellidae	1	0	0	0	0	1	0.5
Spionidae	1	0	0	0	0	1	0.5
**Mollusca**							
**Gastropoda**	7	7	9	21	3	28	14.9
Ampullariidae	0	0	1	1	0	1	0.5
Bithyniidae	2	0	3	6	2	6	3.2
Lymnaeidae	0	3	1	3	0	4	2.1
Physidae	0	0	0	1	0	1	0.5
Planorbidae	0	3	0	4	0	6	3.2
Pleuroseridae	1	0	1	1	0	2	1.1
Pomatiopsidae	0	0	0	1	0	1	0.5
Stenothyridae	1	0	1	0	0	1	0.5
Viviparidae	3	1	2	4	1	7	3.7
**Bivalvia**	4	3	4	8	1	14	7.4
Corbiculidae	2	1	1	1	0	2	1.1
Mytilidae	1	1	1	1	1	1	0.5
Solecurtidae	1	0	0	0	0	1	0.5
Sphaeriidae	0	0	0	1	0	2	1.1
Unionidae	0	1	2	5	0	8	4.3
**Arthropoda**							
**Crustacea**	3	3	3	4	0	8	4.3
Anthuridae	1	0	1	0	0	1	0.5
Aoridae	1	0	0	0	0	1	0.5
Atyidae	0	1	1	0	0	1	0.5
Gammaridae	1	0	1	1	0	1	0.5
Palaemonidae	0	2	0	3	0	4	2.1
**Insecta**	23	22	42	68	18	99	52.7
Caenidae	0	0	1	0	0	1	0.5
Heptageniidae	0	0	1	1	0	2	1.1
Chlorogomphidae	0	0	0	1	0	1	0.5
Gomphidae	0	0	0	2	0	2	1.1
Libellulidae	0	0	0	1	0	1	0.5
Pyralidae	0	0	0	1	0	1	0.5
Aphelocheiridae	0	0	0	1	0	1	0.5
Corixidae	0	0	0	1	0	1	0.5

Chrysomelidae	0	0	1	0	0	1	0.5
Dytiscidae	0	0	1	0	0	1	0.5
Elmidae	0	0	2	0	0	2	1.1
Hydropsychidae	0	0	1	0	0	2	1.1
Polycentropodidae	0	0	1	0	0	1	0.5
Psychomyiidae	0	1	0	0	0	1	0.5
Plecoptera	0	0	0	1	0	1	0.5
Ceratopogonidae	0	0	0	2	1	2	1.1
Chaoboridae	0	0	0	1	0	1	0.5
Chironomidae	23	21	33	54	17	75	39.9
Psychodidae	0	0	0	1	0	1	0.5
Simulidae	0	0	1	0	0	1	0.5
Total	55	47	68	112	45	188	100

Table S3. Abbreviation and full name of assessment indices

Abbreviation	Full name
S_O	observed species richness
S_E	expected species richness
A	Area
O/E-$_{SA}$	observed to expected index based on species-area model
O/E-$_{RF}$ (RIVPACS)	observed to expected index based on random forest model (River Invertebrate Prediction and Classification System)
O/E_0	O/E-$_{RF}$ based on taxa with probabilities of capture greater than 0
O/E_{50}	O/E-$_{RF}$ based on taxa with probabilities of capture greater than 0.5
O/E_0-null	null model of O/E_0
O/E_{50}-null	null model of O/E_{50}
B-IBI	Benthic Index of Biotic Integrity
ASPT	Average Score Per Taxon

Table S4. Abbreviation and description of potential environmental predictors used in modeling

Abbreviation	Description
LON	Longitude
LAT	Latitude
Pour_long	Pour_longitude
Pour_lat	Pour_latitude
AREA*	Lake area (km^2)
Wshd_area	Watershed area (km^2)
Shore_len	Length of shoreline (km)
Depth_avg	Average lake depth (m)
Slope_100	Average slope within a 100-meter buffer around the lake polygon (°)
Dis_avg	Average long-term discharge flowing through the lake (m/s)
Res_time*	Average residence time of the lake water (day/year)
Mois	Average moisture of lake (mm)
SubBas_id	Subbasin identity of lake
ELEV_avg	Average elevation within lake (m)
bio_1	Annual mean temperature (℃)
bio_2*	Mean diurnal range (℃)
bio_3	Isothermality

bio_4	Temperature seasonality (℃)
bio_5	Max temperature of warmest month (℃)
bio_6	Mean of min temperature of warmest month (℃)
bio_7	Temperature annual range (℃)
bio_8	Mean temperature of coldest quarter (℃)
bio_9	Mean temperature of driest quarter (℃)
bio_10	Mean temperature of warmest quarter (℃)
bio_11*	Mean temperature of coldest quarter (℃)
bio_12	Annual precipitation (mm)
bio_13	Precipitation of wettest month (mm)
bio_14	Precipitation of driest month (mm)
bio_15	Precipitation seasonality (mm)
bio_16	Precipitation of wettest quarter (mm)
bio_17	Precipitation of driest quarter (mm)
bio_18	Precipitation of warmest quarter (mm)
bio_19	The precipitation of the coldest quarter (mm)
Prec_mean	Mean precipitation of spring and autumn (℃)
Tmax_mean	Mean of maximum temperature in spring and autumn (℃)
Tmin_mean	Mean of minimum temperature in spring and autumn (℃)
Prec_sd*	The standard deviation of precipitation in spring and autumn (℃)
Tmax_sd	The standard deviation of maximum temperature in spring and autumn (℃)
Tmin_sd	The standard deviation of minimum temperature in spring and autumn (℃)
Prec_cv	The coefficient of variation of precipitation in spring and autumn (℃)
Tmax_cv	The coefficient of variation of maximum temperature in spring and autumn (℃)
Tmin_cv	The coefficient of variation of minimum temperature in spring and autumn (℃)

*Environmental variable selected from RF model in O/E-$_{RF}$

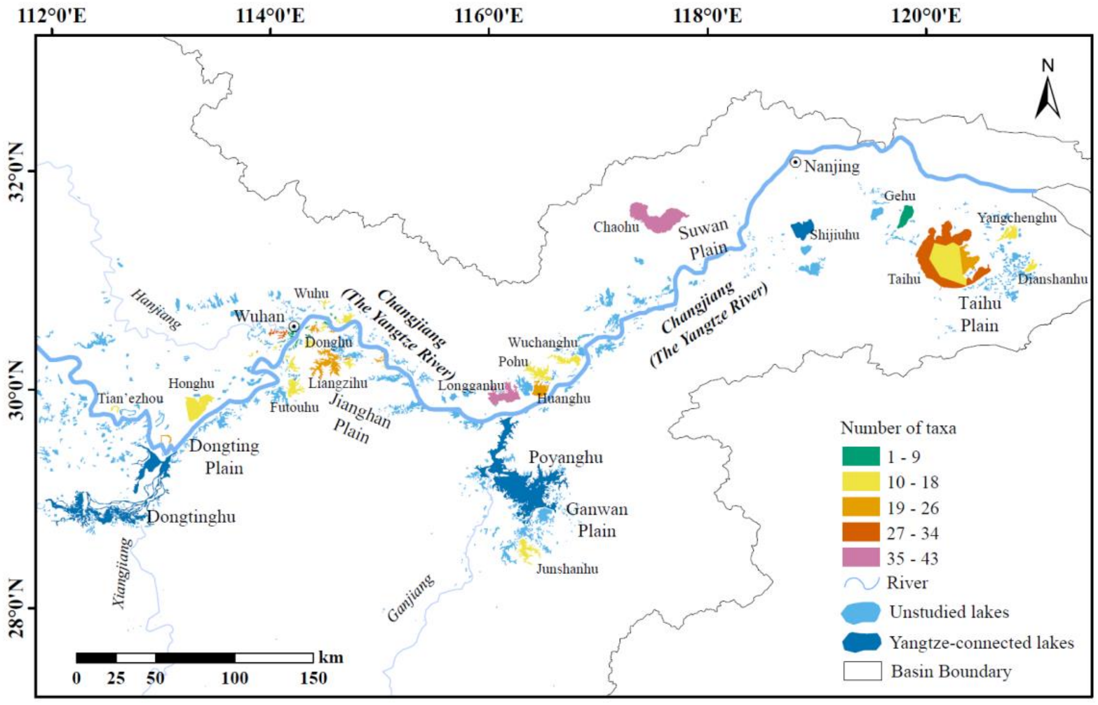

Figure S1. Spatial pattern of macrobenthos of shallow lakes along the mid-lower Yangtze River

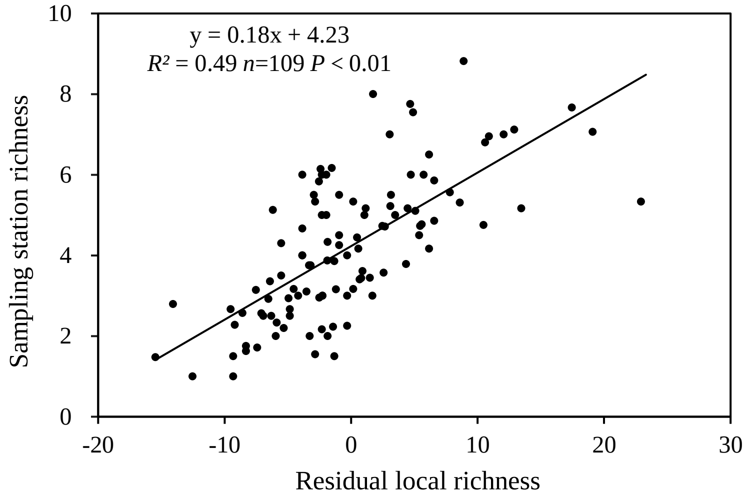

Figure S2. Sampling station vs. residual local taxa richness for macrozoobenthos

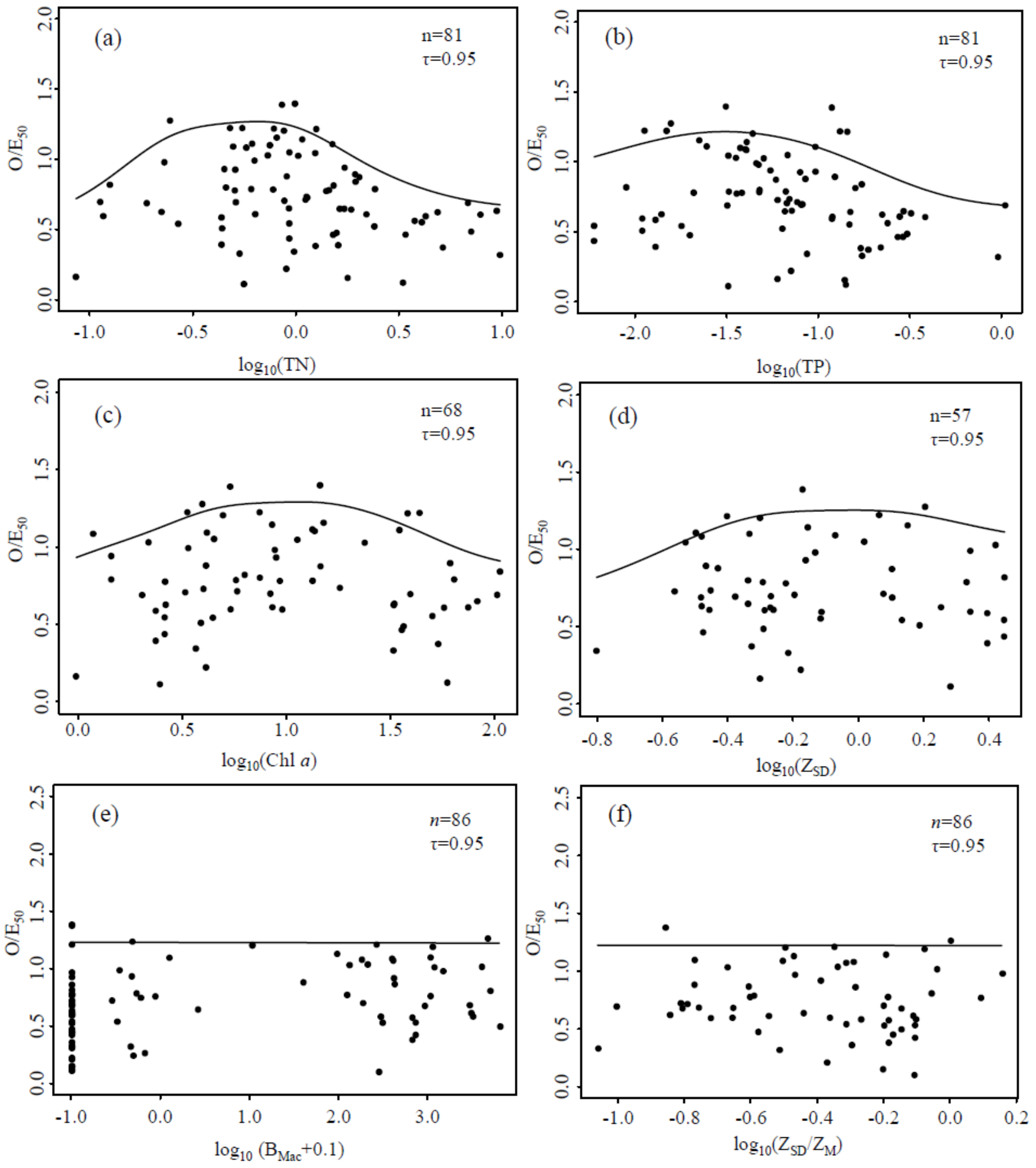

Figure S3. Quantile regressions of O/E_{50} index on total nitrogen (TN, mg/L), total phosphorus (TP, mg/L), chlorophyll *a* of phytoplankton (Chl *a*, μg/L), Secchi depth (Z_{SD}, m), annual submersed macrophytes biomass (B_{Mac}, g/m^2) and ratio of Secchi depth to water depth (Z_{SD}/Z_M, m) (sample sizes differ because of deficient sampling)

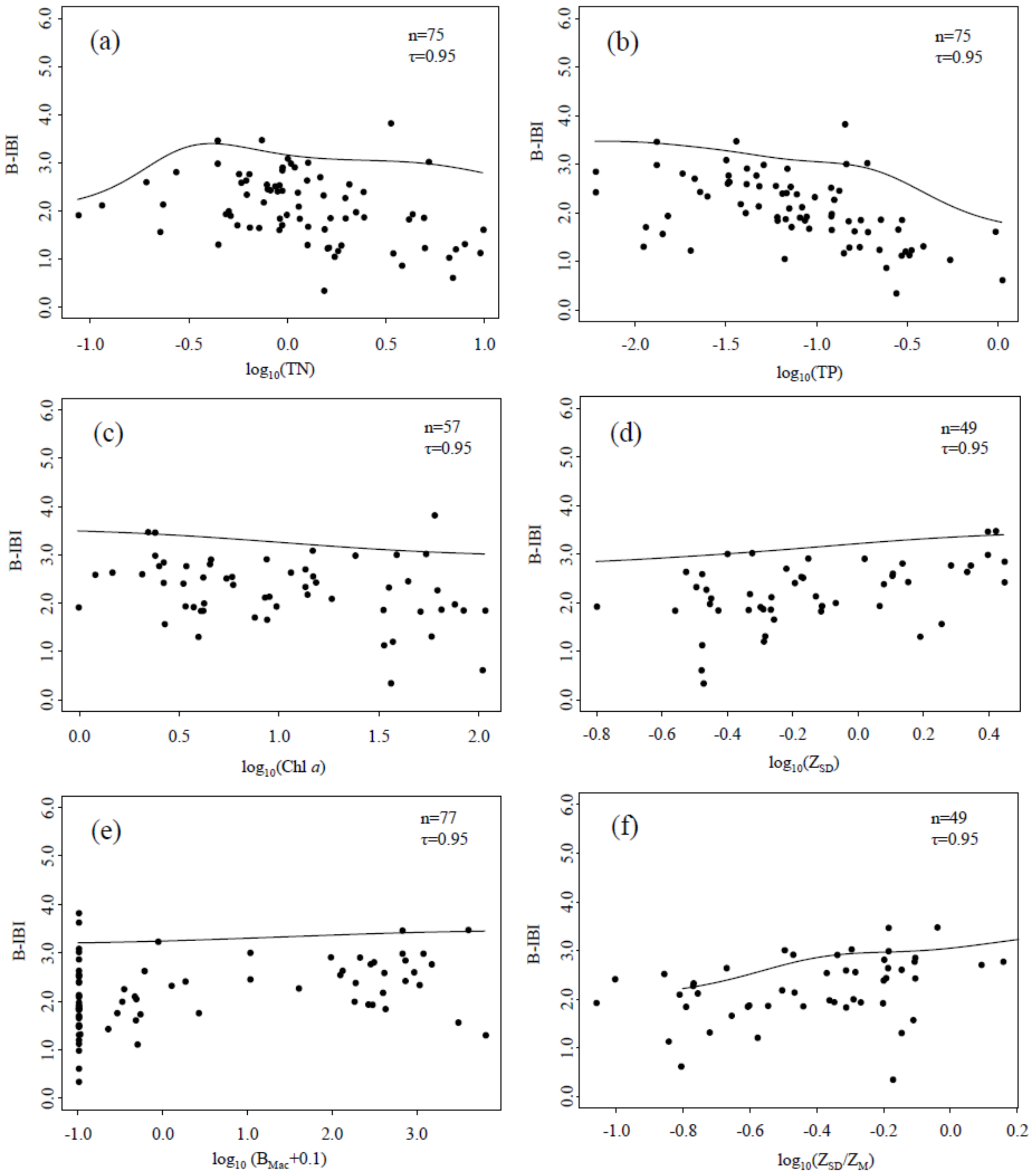

Figure S4. Quantile regressions of B-IBI index on total nitrogen (TN, mg/L), total phosphorus (TP, mg/L), chlorophyll *a* of phytoplankton (Chl *a*, μg/L), Secchi depth (Z_{SD}, m), annual submersed macrophytes biomass (B_{Mac}, g/m^2) and ratio of Secchi depth to water depth (Z_{SD}/Z_M, m) (sample sizes differ because of deficient sampling)

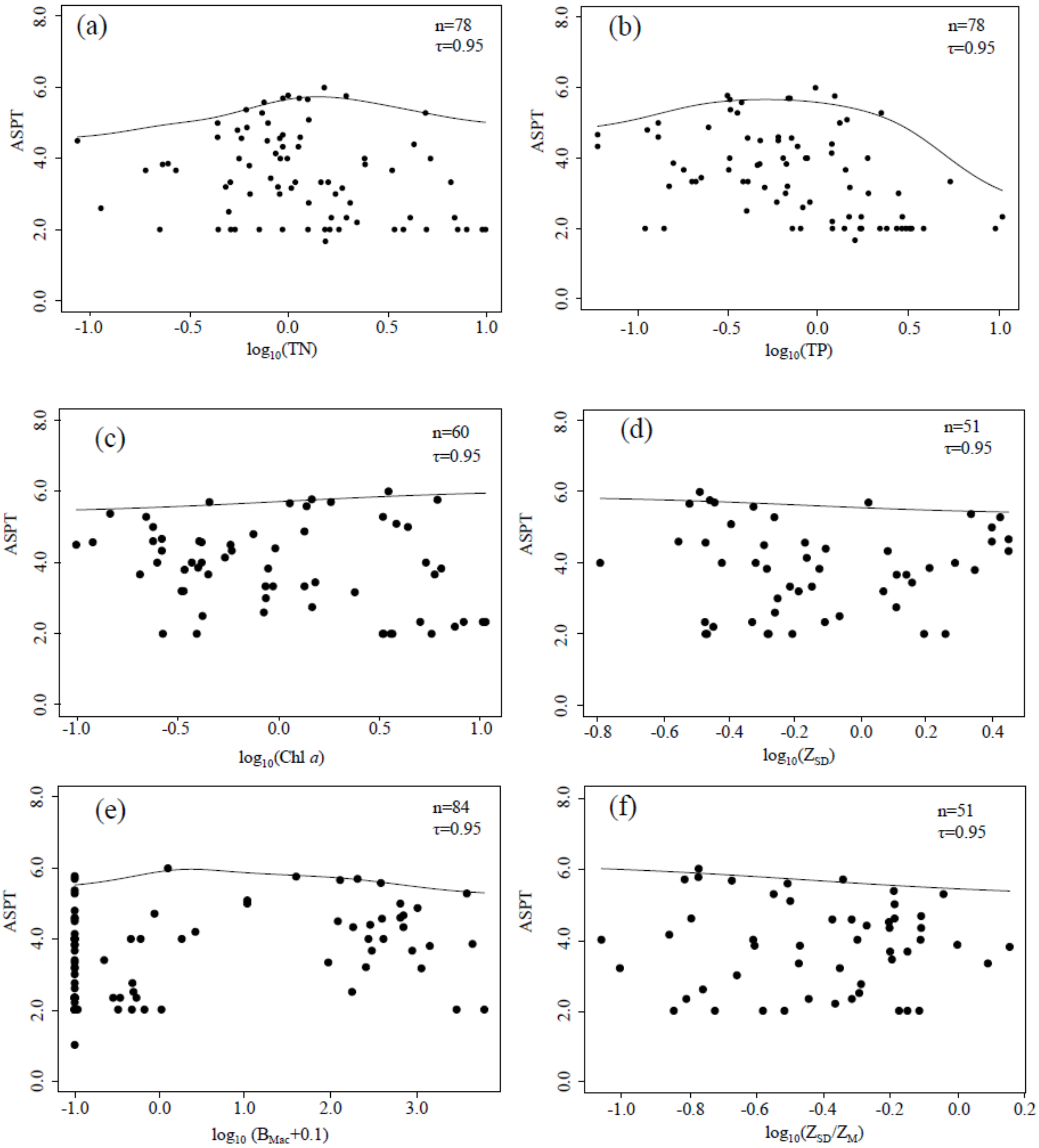

Figure S5. Quantile regressions of ASPT index on total nitrogen (TN, mg/L), total phosphorus (TP, mg/L), chlorophyll *a* of phytoplankton (Chl *a*, μg/L), Secchi depth (Z_{SD}, m), annual submersed macrophytes biomass (B_{Mac}, g/m^2) and ratio of Secchi depth to water depth (Z_{SD}/Z_M, m) (sample sizes differ because of deficient sampling)
